# Sperm Chromatin Immaturity Observed in Short Abstinence Ejaculates Affects DNA Integrity and Longevity *In Vitro*

**DOI:** 10.1371/journal.pone.0152942

**Published:** 2016-04-04

**Authors:** Shubhashree Uppangala, Sherine Eliza Mathai, Sujith Raj Salian, Dayanidhi Kumar, Vikram Jeet Singh, Fiona D’Souza, Guruprasad Kalthur, Asha Kamath, Satish Kumar Adiga

**Affiliations:** 1 Division of Clinical Embryology, Kasturba Medical College, Manipal University, Manipal-576104, India; 2 Department of Community Medicine, Kasturba Medical College, Manipal University, Manipal-576104, India; University Hospital of Münster, GERMANY

## Abstract

**Background:**

The influence of ejaculatory abstinence (EA) on semen parameters and subsequent reproductive outcome is still debatable; hence understanding the impact of EA on sperm structural and functional integrity may provide a valuable information on predicting successful clinical outcome.

**Objective:**

To understand the influence of EA on sperm chromatin maturity, integrity, longevity and global methylation status.

**Methods:**

This experimental prospective study included 76 ejaculates from 19 healthy volunteers who provided ejaculates after observing 1, 3, 5 and 7 days of abstinence. Sperm chromatin maturity, DNA integrity and global methylation status were assessed in the neat ejaculate. Sperm motility, DNA integrity and longevity were assessed in the processed fraction of the fresh and frozen-thawed ejaculates to determine their association with the length of EA.

**Results:**

Spermatozoa from 1 day ejaculatory abstinence (EA-1) displayed significantly higher level of sperm chromatin immaturity in comparison to EA-3 (P < 0.05) and EA-5 (P < 0.01) whereas; the number of 5-methyl cytosine immunostained spermatozoa did not vary significantly across groups. On the other hand, *in vitro* incubation of processed ejaculate from EA-1 resulted in approximately 20 and 40 fold increase in the DNA fragmented spermatozoa at the end of 6 and 24h respectively (P < 0.01–0.001).

**Conclusion:**

Use of short-term EA for therapeutic fertilization would be a clinically valuable strategy to improve the DNA quality. However, use of such spermatozoa after prolonged incubation *in vitro* should be avoided as it can carry a substantial risk of transmitting DNA fragmentation to the oocytes.

## Introduction

Earlier studies have clearly shown that duration of the ejaculatory abstinence (EA] influences semen parameters such as semen volume and concentration [1–3], while change in motility pattern in relation to the length of abstinence is still contradictory [3–4]. As the reports on the influence of EA on semen parameters and subsequent reproductive outcome are still debatable, understanding the impact of EA on sperm structural and functional integrity may provide valuable information on counselling infertility patients [5].

The recommendation for 3–7 days of EA prior to any therapeutic procedures such as intrauterine insemination (IUI) and assisted reproductive technology (ART) has been based on the fact that prolonging EA interval maximizes the sperm concentration in the ejaculate [6–8]. Interestingly, an earlier study has shown that a short period of EA before intrauterine insemination (IUI) is associated with higher pregnancy rates despite a lower sperm concentration in the ejaculate [9].

Approximately 85% of histones in sperm DNA are replaced by protamines. Though, protamination takes place during spermiogenesis in the seminiferous tubules, epididymal environment does play a role in stabilizing the chromatin architecture [10–11] possibly to facilitate the timely decondensation and also to prevent premature degradation of sperm DNA. Sperm with abnormally low protamine level have a higher retention of histone proteins [12], which eventually make the sperm vulnerable to genetic and epigenetic errors [13–14]. However, the association of sperm chromatin maturity and integrity with the longevity in relation to the length of EA is not elucidated so far. We hypothesized that shorter EA may compromise the longevity of sperm DNA under *in vitro* conditions due to inadequate time available for epididymal programing of the spermatozoa though; studies have suggested that good DNA bearing sperm can be enriched by a short abstinence period [15–16]. Therefore, the specific aim of the study was to understand the association between sperm chromatin maturity and global methylation status, sperm survival, DNA integrity, and its longevity in relation to the length of the EA.

## Materials and Methods

### Study subjects

This prospective study included 76 ejaculates from 19 healthy volunteers. Kasturba Hospital Institutional Ethics Committee approval was obtained (IEC 247/2013) before the initiation of the study. Written informed consent was taken from all the subjects who participated in this study. Each volunteer was requested to produce a fresh semen sample after observing the abstinence period of 1, 3, 5 and 7 days.

### Semen analysis

Semen analysis was performed within 1 h of collection under sterile conditions. Upon liquefaction, the sample was mixed well and evaluated for basic semen parameters according to world health organization [8] guidelines. Semen parameters such as semen volume, concentration, motility, morphology and vitality were recorded. Viability was determined by Eosin-nigrosine staining. Sperm morphology was assessed after Papanicolaou’s staining according to WHO (2010) guidelines. All the above parameters were assessed by two independent observers.

### Aniline blue staining

Sperm chromatin maturity was assessed according to Terquem and Dadoune [17] with minor modification. A thin smear of the ejaculate was prepared on a glass slide, and allowed to dry. The smears were fixed for 30 min in 3% glutaraldehyde in phosphate buffered saline (pH 7.2) and stained with 5% aqueous Aniline blue prepared in 4% acetic acid (pH 3.5) for 4 min. Slides were rinsed in water, counter stained with 0.5% Eosin for 3 min and mounted using DPX. A minimum of 500 spermatozoa from each sample were evaluated and the percentage of aniline blue positive heads was calculated in order to determine the percentage of spermatozoa with immature chromatin.

### Immuno detection of 5-methyl cytosine (5mC)

Immunostaining was performed according to the previously described method [18]. The slides containing spermatozoa were air dried followed by fixation in 4% paraformaldehyde (PFA) for 20 min. The cells were washed in PBS and decondensation was done in 25mM DTT followed by denaturation with 6N HCl. The spermatozoa were treated with monoclonal anti-5-methylcytosine (5mC) antibody (Cat. No. NA81, Calbiochem) at a dilution of 1: 50 and incubated overnight at 4°C in a humidified chamber. After washing with PBS, the cells were incubated with FITC labeled Goat anti-mouse IgG (Cat. No. D0408, Santa Cruz), at a dilution of 1:200 for 1h at 37°C. The cells were counterstained with propidium iodide (PI) and observed under fluorescence microscope (Imager-A1, Zeiss, Germany) at 100X magnification. Spermatozoa fluorescing green were considered as hypermethylated. Minimum of 2,000 spermatozoa were scored from each data point from two independent observers. If the differences between two readings are >10%, then repeat analysis was performed on a particular sample.

### Semen cryopreservation-thawing

Ejaculates were cryopreserved using SpermFreez^TM^ (FertiPro, Belgium) in a single step freezing medium according to the manufacturer’s protocol. Samples were maintained at -196°C until thawing.

### Survival and DNA longevity analysis

Spermatozoa from the fresh and frozen-thawed ejaculates were washed in Earle’s balanced salt solution (EBSS) supplemented with 0.1% human serum albumin using swim-up technique. The processed fraction of the spermatozoa was suspended in 200μL EBSS medium, incubated at 37°C and 5% CO_2_. The sperm motility and DNA longevity was determined at 0h (as soon as swim up fraction was collected), 1h, 6h and 24h. In case of frozen thawed ejaculates, 0 and 6h intervals were used for motility and DNA longevity assessment.

### Sperm chromatin integrity analysis

Sperm chromatin dispersion (SCD) assay was performed in fresh ejaculate, frozen thawed ejaculate as well in washed spermatozoa from fresh and frozen thawed group at different intervals as described by Fernandez *et al*. [19] with minor modifications [20]. Briefly, the spermatozoa were mixed with 1% low melting point agarose maintained at 37°C. About 150μL of this mixture was layered on a glass slide pre-coated with 0.65% of normal melting point agarose and the gel was allowed to solidify. The slides were immersed in freshly prepared acid denaturation solution (0.08 N HCl) for 7 min at room temperature in dark. Proteins were removed by incubating the slides in lysing solution 1 containing 0.4M Tris, 20mM DTT, 1% SDS, 50mM EDTA, at pH 7.5 for 20 min followed by incubation in lysing solution 2 containing 0.4M Tris, 2M NaCl, at pH 7.5 for 15 min at room temperature. Neutralization was done in Tris buffer (0.4M Tris, pH 7.5) for 2 min, serially dehydrated in graded ethanol, and air dried. Cells were stained with Ethidium Bromide (2μg/mL) and sperm chromatin integrity was assessed in a minimum of 400 spermatozoa under fluorescent microscope (Imager- A1 Carl Zeiss, Germany). The percentage of sperm with chromatin damage was determined by counting the spermatozoa having fragmented nuclei and spermatozoa with no halo and small halo.

### Statistical analysis

The data represents mean and standard deviation (Mean ± SD) of the values. All the data points were tested for normality. The difference between the groups was determined using Repeated measures ANOVA with Bonferroni post hoc test. A P value < 0.05 was considered statistically significant. The data were analyzed using the Statistical Package for the Social Sciences for windows (SPSS 20; SPSS, Inc., Chicago, IL, USA). The graphs were plotted using Microcal Origin 6 (Origin lab, Northampton, USA) and SigmaPlot 13 (Systat software Inc. California, USA)

## Results

All the 19 subjects included in this study strictly complied with abstinence requirements.

Semen characteristics of the subjects in relation to length of EA are presented in Table 1 and S1 Table. Both semen volume and concentration showed a positive association with respect to duration of the abstinence (P < 0.01). However, no significant variation was observed in the number of morphologically normal and viable spermatozoa though decreasing trend was seen with the increase in the length of EA.

**Table 1:** Basic semen characteristics in relation to different EA intervals.

Parameters (Mean±SD)	EA -1	EA -3	EA -5	EA -7
Semen Volume	2.1±0.77 ^a^	2.71±1.37 ^a^	3.25±1.37	3.52±1.30
Total Sperm Number	111.3±83.56 ^b^	177±125.9	224.3±137.7	239.06±166.8
Total Motility (%)	72.52± 16.25	71.26±8.58	64.1±17.45	66.21±14.14
Viability (%)	67.6±8.09	67.1±13.46	60.7±13.97	54.4±15.41
Normal Morphology (%)	38.63±10.25	38±12.02	35.27±11.67	34.45±11.32

^a^ P < 0.01 Vs EA-5 and EA-7

^b^P < 0.01 Vs EA-5 and EA-7.

### Increased level of sperm chromatin immaturity is related to short EA

**Fig 1:**
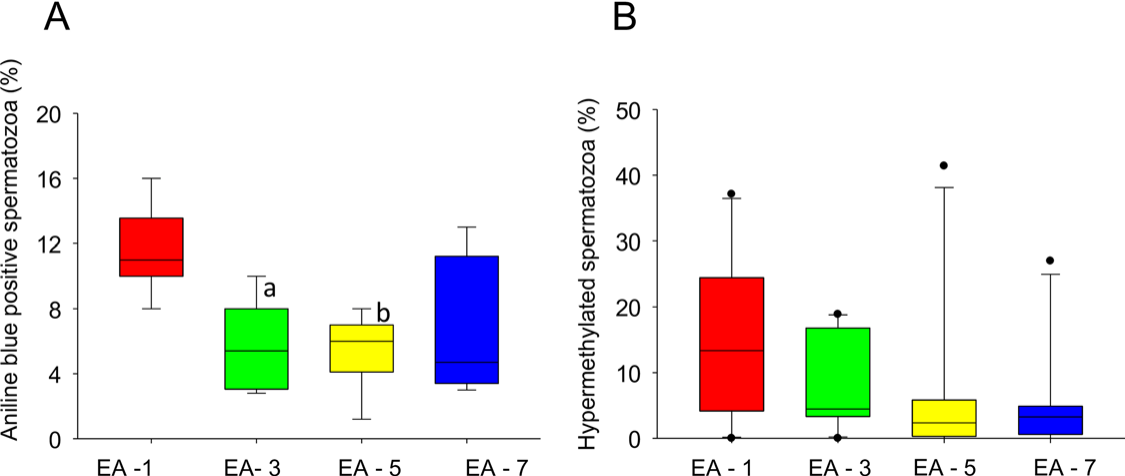
Sperm chromatin maturity and hypermethylation level at various EA periods. **A)** Box plot demonstrating the percentage of aniline blue positive spermatozoa (suggestive of immature chromatin) in the neat ejaculate from four study intervals (N = 32) (^a^ P < 0.05 *Vs* EA-1, ^b^ P < 0.01 *Vs* EA-1). **B)** Box plot demonstrating the percentage of 5mC positive spermatozoa (suggestive of hypermethylation) across study intervals (N = 40). Please note that the differences were not statistically significant.

Analysis of sperm chromatin maturity by aniline blue staining has demonstrated almost two fold increase in the percentage of aniline blue positive spermatozoa in EA-1 interval which was significantly higher in comparison to EA-3 and EA-5 intervals (P < 0.05–0.01) (Fig 1A and S2 Table). This observation suggests that short epididymal transit period may have a role in sperm chromatin maturity. To understand whether this spermatozoal immaturity has any influence on epigenetic programing, the global methylation level was studies by 5mC immunostaining in the neat ejaculate of different EA intervals. Since microscopic immuno detection of 5mC is the crudest method for methylation analysis in sperm cells, maximum precaution was taken during various steps of staining to minimize the technique related variations. Cells which did not stain by 5mC antibody but stained by nuclear stain were considered as ‘hypomethylated’ whereas cells stained by 5mC irrespective of intensity of staining were considered as ‘hypermethylated’. The percentage of hypermethylated spermatozoa was higher in EA-1 interval in comparison to the other intervals. However, the differences were not statistically significant (Fig 1B and S2 Table).

### DNA longevity is compromised in the processed spermatozoa from short EA

**Fig 2:**
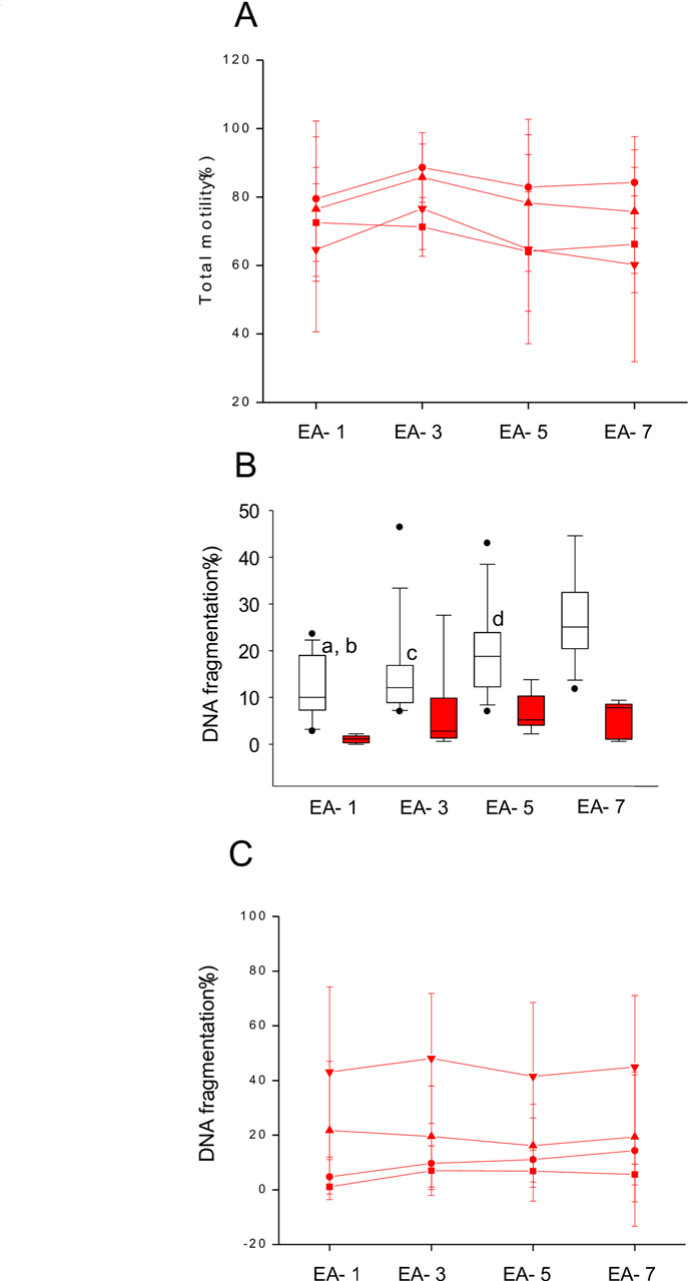
Sperm motility and DNA fragmentation analysis in relation to EA. A) Sperm motility analysis in neat ejaculate (N = 76) (■) as well as in the processed fraction (N = 32) from four study intervals. Processed fraction was incubated at 37°C and motility analysis was performed at 0h (●), 6h (▲), and 24h (▼) time interval. Please note that differences in sperm motility with corresponding EA intervals were not statistically significant. B) Box plot depicting the DNA fragmentation level as measured by the sperm chromatin dispersion (SCD) assay in the neat ejaculate (N = 56) (□) and processed fraction (N = 32) (■). ^a^P < 0.05 *Vs* corresponding group in EA-5; ^b^P < 0.001 *Vs* corresponding group in EA-7; ^c^ P < 0.01 *Vs* corresponding group in EA-7; ^d^P < 0.05 *Vs* corresponding group in EA-7. C) Sperm DNA longevity analysis by SCD assay in the processed fraction at 0h (■), 1h (●), 6h (▲), and 24h (▼) time interval.

Sperm motility in EA-1 interval was comparable to other intervals. In addition, the assessment of sperm motility at various post wash time intervals did not show any association with the length of EA (Fig 2A and in S3 Table).

Sperm DNA fragmentation was assessed by sperm chromatin dispersion test in both neat ejaculates and processed sperm fractions of various EA intervals. We observed an intra-individual random variation in the number of DNA fragmented spermatozoa in relation to different EA (S4 Table). However, repeated measures analysis has demonstrated significant differences between study groups. Fig 2B (and S4 Table) depicts DNA fragmentation level in spermatozoa from various EA periods where EA-1 had significantly lower DNA fragmentation compared to EA-5 (P < 0.05) and EA-7 (P < 0.001). Similarly EA-3 and EA-5 had significantly lower level of DNA fragmentation compared to EA-7 (P < 0.01 and P < 0.05). Processing the ejaculates by swim up technique significantly eliminated the number of DNA fragmented spermatozoa in all the EA intervals (P < 0.05–0.0001). It is interesting to note that almost 10-fold decrease in the number of DNA fragmented spermatozoa was observed in the processed fraction of EA-1 whereas the reduction was only 2–5 folds in other EA intervals.

Sperm DNA longevity was assessed at various time intervals in the processed fraction of the ejaculates until 24 h to understand the association between DNA stability and duration of the EA. The extent of DNA fragmentation did not vary significantly across the intervals at various time periods tested. Interestingly, 6h *in vitro* incubation of processed ejaculate at 37°C resulted in approximately 20.2 fold increase in the number of DNA fragmented spermatozoa in the EA-1 interval (P < 0.001). However, the increase was only 2.8, 2.4 and 3.5 fold in EA-3, EA-5 and EA-7 intervals respectively. Importantly, the DNA fragmentation level was increased further by approximately 40.1 fold in EA-1 at 24h (P < 0.01) whereas the increase was only 6.9, 6.1 and 8.0 fold in EA-3, EA-5 and EA-7 intervals respectively (Fig 2C and S5 Table). These results suggest that spermatozoa isolated from the short ejaculatory abstinence (EA-1) have higher tendency to undergo DNA fragmentation *in vitro*.

### Freeze-thaw process does not affect DNA integrity in short abstinence ejaculates

**Fig 3:**
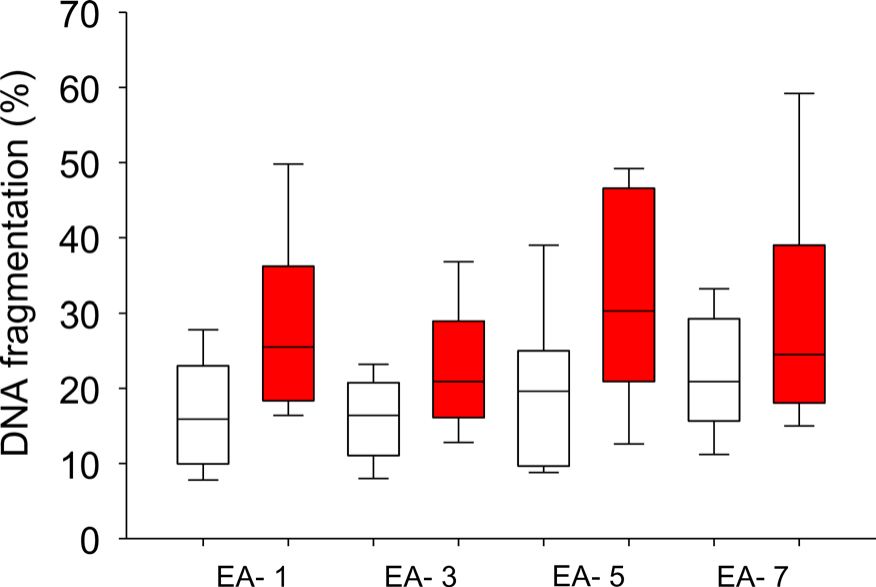
DNA fragmentation analysis in frozen thawed spermatozoa. DNA fragmentation level was measured by the sperm chromatin dispersion (SCD) assay in the processed fraction at 0 (N = 32) (□) and 6h (N = 32) (■). Please note that fold change in the sperm DNA fragmentation level with corresponding interval of EA were not statistically significant.

*In vitro* enhancement in DNA fragmentation in the short term abstinence ejaculates prompted us to look into the effects of freeze-thaw process on sperm DNA fragmentation and its relation to the length of EA. A moderate non-significant increase in the level of DNA fragmentation was observed in the post-thaw ejaculates and the differences were not significantly different across the EA intervals. *In vitro* incubation of processed post-thaw ejaculates until 6h showed approximately 1.3 to 1.7 fold increase in the level of DNA fragmentation. Unlike freshly processed ejaculates (Fig 2C), frozen-thawed, processed spermatozoa from EA-1 did not show any significant difference in the level of DNA fragmentation compared to other EA intervals (Fig 3 and S6 Table).

## Discussion

The results presented in this study have important clinical implications. First, the number of spermatozoa with chromatin immaturity was significantly higher in EA -1 compared to other groups. However, it was noteworthy that a significant number of spermatozoa in this interval had intact DNA which was further enriched by the swim up technique. On the other hand, results have also suggested that spermatozoa isolated from EA-1 have higher tendency to undergo DNA fragmentation over an extended period of time *in vitro*.

During spermiogenesis, majority of histones are replaced by sperm specific protamines which changes somatic type chromatin into supercoiled structure [21]. The epididymal transit further strengthens the chromatin structure of the spermatozoa by cross-linking of protamines by disulphide bonds. The unique densely compacted chromatin architecture protects the mature spermatozoa against exogenous toxic assault [22]. However, a decreased time frame between each ejaculation speeds up the passage of spermatozoa in the epididymis and as a consequence spermatozoa are not adequately exposed to the conducive epididymal environment for post testicular chromatin modification [23] which could explain the poor sperm chromatin maturity observed in EA-1 interval.

Aberrant sperm DNA methylation has been associated with fertility disorders in men thus methylation analysis has been considered as a marker of testicular function and spermatogenesis [14]. Though, we observed an increase in the number of hypermethylated spermatozoa in EA-1 group, statistical power was not established due to intra individual random variation in the number of hypermethylated spermatozoa with different EA (S2 Table). At this juncture, it is important to note that, global DNA methylation analysis of human sperm cells using 5mC immunostaining may not be capable of detecting subtle epigenetic variations in individual gene regions. Hence our observation highlights the need for further analysis of specific imprinted genes to elucidate the association between methylation status and EA.

Taken all together, our findings suggest that chromatin immaturity in the ejaculates could be related to their short epididymal transit as the differences were evident in EA-1 interval. These observations highlight the need for further analysis of functional and genetic integrity of the spermatozoa derived from short EA. Earlier reports are supportive of our observation that motility patterns remain unaffected by the duration of abstinence [24]. Apart from motility analysis in the ejaculate, extended motility assessment in the processed fraction until 24h also did not demonstrate any significant association with the duration of abstinence. On the other hand, sperm DNA fragmentation in EA-1 interval was significantly low which is in agreement with a previous report where the ejaculate from 24 h abstinence had increased number of DNA intact spermatozoa [15]. We also analyzed, for the first time the efficiency of swim up technique in extracting the DNA intact sperm in relation to the duration of abstinence. Interestingly, almost a 20-fold decrease in the number of DNA fragmented spermatozoa was observed in the processed fraction of EA-1 interval whereas the reduction was only 2 to 5 fold in other EA intervals. This observation suggests that DNA fragmented spermatozoa can be effectively eliminated from the ejaculate with 1 day abstinence by routinely used sperm wash techniques.

Of course, applying 1 day EA and swim up extraction of DNA intact spermatozoa could be a clinically valuable strategy to reduce the impact of fertilization by DNA fragmented sperm. However, the poor chromatin maturity observed in EA-1 interval prompted us to look into the longevity of sperm DNA as the chromatin architecture and maturity of spermatozoa tend to affect their decondensation capability *in vitro* [22]. To our surprise, about 40 fold increase in the DNA fragmentation level was observed in the processed spermatozoa from EA-1 interval at the end of 24h of *in vitro* culture. It is possible that immature sperm produced after recurrent ejaculation is vulnerable to undergo DNA fragmentation due to limited time span available within the epididymis for strengthening the chromatin structure, thus producing large differences in DNA fragmentation between EA-1 and rest of the intervals studied.

Since semen cryopreservation is known to affect the sperm chromatin structure [25], we were interested to see the influence of abstinence related chromatin immaturity on post thaw sperm DNA integrity and its longevity. In contrast to the poor DNA longevity of freshly processed fraction, frozen thawed spermatozoa from EA-1 interval did not show significant difference in the DNA fragmentation until 6h of *in vitro* incubation.

Earlier studies have suggested that 1–2 days of EA is associated with a higher pregnancy rates following IUI although the number spermatozoa available for insemination in these intervals were few [9]. Our findings do indicate that 1 day EA is useful in recovering DNA intact sperm fraction for the therapeutic purposes. However, we must bear in mind that the use of such spermatozoa for fertilization after prolonged incubation does carry a substantial risk of transmitting DNA fragmentation to the oocytes and embryos. Further studies are essential to understand the fertilization, developmental and implantation potential of the embryos derived from EA-1 spermatozoa.

The strength of our study is that a single subject has provided ejaculates at multiple intervals. In addition, DNA longevity was assessed in relation to the EA. However, the limitation was that only normozoospermic ejaculates were used and from the clinical point of view, it is important to understand the DNA longevity of spermatozoa from abnormal ejaculates as poor quality ejaculates carry higher level of chromatin abnormalities in spermatozoa [26] and spermatozoa from infertile patients exhibit considerably higher DNA instability than normal semen donors [27]. In conclusion, use of short-term EA for therapeutic fertilization would be a clinically valuable strategy to improve the DNA quality. However, use of such spermatozoa after prolonged incubation *in vitro* should be avoided as it can carry a risk of transmitting DNA fragmentation to the oocytes. Overall these findings may help clinicians to determine the best treatment options for a couple. For example, ICSI does not require DNA longevity while IUI does.

## Supporting Information

S1 Table Basic semen characteristics in relation to various EA intervals. (XLSX)

S2 Table Percentage of aniline blue positive spermatozoa and percentage of 5-mC positive spermatozoa at various EA periods. (XLSX)

S3 Table Total motility in the processed fraction of the ejaculate from various EA intervals. (XLSX)

S4 Table Sperm DNA fragmentation in fresh and processed ejaculate (0h) at various EA intervals. (XLSX)

S5 Table DNA longevity analysis by SCD assay in spermatozoa from various EA intervals. (XLSX)

S6 Table DNA fragmentation in the processed fraction of frozen thawed spermatozoa. (XLSX)
